# Salmagundi

**Published:** 1885-04

**Authors:** E. G. Betty


### SALMAGUNDI.

EDITED BY E. G. BETTY, D. D. S.

A school boy in Lewiston, Me., says the Journal of that place, being given the word "toward" to construct a sentence with, wrote on the blackboard, "I toward my trousers."

Jefferson Hayes, a colored gentleman of Hawkinsville, Ga., is the champion stump puller, having removed 827 stumps from thirteen acres. The close connection between stumps and acres suggests the possibility of a good pun for some suffering paragrapher.—*Daily Paper.*

---

A WESTERN exchange contains an advertisement from an undertaker wanting a partner, and winds up with the injunction that "only a live man need apply." The coffin-maker evidently did not want to hear from any of those with whom he had formerly done business.—*Boston Gazette.*

---

THE Medical Department of the Columbian University, Washington, D. C., has opened its doors to women during the present session, four of whom have already matriculated. This step was not taken without due deliberation, and the male students do not seem to be disturbed by the introduction of a new element into their midst.

---

FLUX.—Cut into small pieces half a pound of sheet zinc, and place in an earthen jar, then pour one pound of muriatic acid; let it boil half an hour, then add half an ounce of muriate of ammonia, quarter of an ounce of borax; when that is dissolved, add half a pound of rain water, and strain. Thus solder may be used by melting with the blow-pipe, a soldering iron, or an alcohol lamp.

---

DISTINCTIONS CONFERRED UPON DENTISTS.—Dr. Miller, whose name is well known in connection with the germ theory of dental caries, has been appointed by the German Minister of Education, Royal Professor in the University of Berlin. This is an honorable distinction of notable value, and is conferred only upon those whose work is held in high esteem by the powers that be.

---

A GENERATION ago there were many who resented the supposition that man had sprung from the ape. But on reflection most of us have discerned that this repugnance came rather from pride than wisdom, and that with the race, as with the individual, there is more true hope for him who has risen by education from the beggar boy than for him who has fallen by transgression from the prince.—*Contemporary Review.*

---

THE dental school and hospital of Paris celebrated ts fifteenth year early in November. M. Paul Bert presided. In his speech he criticised adversely the doctrine that all dentists should obtain a doctor's degree. Dental students should be required to have an exact and extended knowledge of all subjects in connection with their profession. The President terminated his address by expressing a hope that the French dentists would soon possess a college equal to that of their American colleagues.

---

CHROMIC ACID IN AFFECTIONS OF THE MOUTH.—Dr. Cauquil writing to the *Bulletin General de Therapeutique*, concerning the employment of chromic acid, as recommended by Dr. Butlin in diseases of the tongue, states that he has also found it of value in stomatitis. He has used it with success in mercurial sore mouth, in the strength of one to five. In other cases of syphilitic pharyngitis, with hypertrophy of the tonsils, he has obtained good results with the same solution.

---

M. MICHEL, a medical man practicing at Chaumont, in a note to the Academie des Sciences of Paris, publishes evidence confirming the accuracy of M. Marey's communications concerning the part played by water in spreading epidemics. It appears that every year there were a considerable number of deaths at Chaumont from typhoid fever. Recently, a water supply, which was believed to be contaminated, was cut off; since that time, typhoid has disappeared.

---

INTEMPERATE EATING.—Two cases are reported in an exchange. The first is that of a railway clerk, who appeared well when he went to bed on Sunday night, but died before morning. The medical man who examined the body found the stomach largely distended with undigested food, which had stopped the action of the heart. The other case was that of a negro from Sierra Leone in whose stomach whole potatoes were found.—*Druggist's Circular.*

The Tooth-Pick Business.—The caricaturist of the future may perhaps represent the typical Yankee no longer as whittling, but going through the process called "chewing the quill." In nearly every hamlet, town, and city of the country the consumption of the tooth pick, both physically and commercially, has become a national characteristic. Few, indeed, are the hotels, restaurants, etc., where the invitation to "have a "toothpick" is unknown; while no private house is considered well ordered in the absence of this edible. It has even invaded the domain of literature, and we are no longer supposed to "chew the cud," but the quill "of reflection." For this general result a firm in this city is largely responsible, as the introduction of the wooden toothpick was made by it. Said a member of the firm to a Tribune reporter:

"We began to make wooden tooth-picks fifteen years ago, and while another company now share the wholesale trade, we were the sole manufacturers of the article ten years. We have a white wood kind made of poplar, and a hard wood kind made of birch. The picks are made in Maine, the lumber of that state supplying the material. Sometimes we flavor picks, say with cinnamon or wintergreen. What does the trade amount to in wooden picks? Well, I should say about twelve thousand cases a year, one thousand of which is exported to Europe and Mexico. A case contains one hundred boxes and each box has twenty-five hundred picks. So, you see, there's billions in it. Has the business increased much? About twenty-five per cent in the last three years. Orange wood picks, imported, are to be found in some clubs, and also a flavored wood-pick from Japan. Quills are wholly imported. Russia and Sweden furnish the material, and these countries with France and Germany also manufacture the article as we get it. The work is done altogether by hand. Peasant folks, after their daily work is done, sit down with a sharp knife and add to their pin money by cutting these quills. This adds to their price here, of course. Perhaps some inventive American will construct a machine some day that will turn them out rapidly and make them as popular as the wooden picks now seem to be.—*New York Tribune.*

Echoes of the M. V. D. A.—The forty-first annual meeting of the Mississippi Valley Dental Association was a grand success and no mistake. The attendance was the largest for five or six years past and the interest was intense. No fewer than ten papers were read ; Drs. Arnold, Berry, Porre, Kempton, Wright, Harlan, Cassidy, Rawls, Van Antwerp, and G. W. Smith each reading one. In addition to these there remained four unread as the reading and discussion of the above consumed the time up to adjournment. These remaining papers by Drs. Billings, Sage, Callahan, and Betty will be published with the rest. It is far better that there should be a surplus of papers than that there should be no papers at all as has so often been the case of late. It would not be a bad idea for the members of the M. V. D. A. to buy extra copies of the Register, which will contain all the essays, discussions, and proceedings of the meeting, and have them bound seperately and labeled "Tranactions of the Forty-first Annual Meeting of the M.V. D. A." That would look so nice, and be a novelty we have not yet had the pleasure of possessing besides being in a form for ready reference. The business of this meeting will probably occupy three numbers of the "Register."

---

Dr. H. J. McKellops, of Saint Louis (and there is only *one* McKellops) was present and took a most active part in the discussions much to the pleasure of his many friends in the M. V. D. A., who have not of late heard his voice lifted up in earnest discourse.

---

The papers of Drs. Harlan and Cassidy on "New Remedies for the Treatment of Dental and Oral Diseases," together with the discussions thereon, were alone worth a long journey to hear, and all those who were absent will feel sorry they were not present at this meeting.

---

Dr. Watt, though out of sight, was not out of mind for the dear old M. V. D. A. sent her kindest regards to him, one of her first born ; what mother ever forgets an afflicted son ?

The impression seems to be gaining ground, that it would be much better for the M. V. D. A., to visit about among the cities of the Mississippi Valley. This is a question that is worthy of consideration and deliberation, and we cordially invite the readers of the Register and they are largely members of the society, to give "Salmagundi" their opinion upon the subject and see what is best to be done about it.

